# Bell-bottom technique in iliac branch era: mid-term single stent graft performance

**DOI:** 10.1186/s42155-020-00147-w

**Published:** 2020-11-15

**Authors:** Gabriele Pagliariccio, Emanuele Gatta, Sara Schiavon, Carlo Grilli Cicilioni, Simona Lattanzi, Elisa Dimitri, Luciano Carbonari

**Affiliations:** 1 Department of Vascular Surgery, Azienda Ospedaliero Universitaria Ospedali Riuniti Ancona, Via Conca, 71, 60126 Ancona, Italy; 2 Department of Experimental and Clinical Medicine, Clinic of Neurology, Marche Polytechnic University, Ancona, Italy

**Keywords:** Common iliac artery aneurysm, Bell-bottom technique, Iliac branch device, Type 1b endoleak

## Abstract

**Background:**

Endovascular abdominal aortic aneurysm repair (EVAR) is considered the primary option for abdominal aortic aneurysm but the management of concomitant wide or aneurysmal iliac arteries (CIAs) is still controversial.

**Methods:**

We retrospectively evaluated mid-term results of patients receiving standard EVAR combined with bell-bottom technique (BBT) using Medtronic Endurant endograft between January 2009 and December 2018. Patients were followed up by CT scan performed 1 month after the procedure and by duplex ultrasound annually (with or without contrast medium) followed by CT scan in case of evolution.

**Results:**

Seventy-one patients (67 males; mean age of 77,1 years) with abdominal aortic aneurysm and wide or aneurysmal common iliac artery (distal landing zone diameter up to 25 mm and length more than 20 mm) were treated with standard EVAR and BBT (107 limbs) using Endurant stent graft. The median aortic diameter was 56,1 mm (31.0-85.0). Technical success was obtained in 100%. Mean procedural time was of 100.1 min. No 30 days’ mortality, renal failure or limb ischaemia occurred. The median follow-up was of 36.56 months (1–136). 5-year aneurysm related mortality was not found. At 5 years, the number of all-cause deaths was seven. The freedom from secondary intervention was 91.6% at 5 years. Three patients (4.4%) were treated for iliac related complications at 5 years: internal iliac artery aneurysm, iliac obstruction, type 1b endoleak, all successfully treated by endovascular technique.

**Conclusions:**

According with this study BBT using Endurant stent graft is effective and safe with good mid-term results, with low rate of iliac related complications and no aneurysm related mortality.

## Introduction

Currently in most patients with suitable anatomy and reasonable life expectancy, endovascular abdominal aortic aneurysm repair (EVAR) is considered the primary treatment option (Wanhainen et al. 2019); however, the management of concomitant wide or aneurysmal iliac arteries (CIAs) is still controversial.

Large iliac arteries are reported to have a higher risk of type 1b endoleak by some authors (Wanhainen et al. 2019). Further, there are some reports of secondary dilatation of the distal landing zone over time resulting in a type 1b endoleak, especially when the common iliac arteries are wide or aneurysmal.

Multiple technical solutions (bell-bottom technique, iliac branch devices, landing in the external iliac artery with concomitant internal iliac artery (IIA) occlusion, surgical replacement or endovascular revascularization of the internal iliac artery with “banana technique” or parallel graft) have been proposed (Massière et al. 2016) but a clear algorithm is still lacking.

The bell-bottom technique (BBT) has been used for many years in patients with abdominal aortic aneurysm (AAA) and concomitant wide or aneurysmal CIA with distal landing zone diameter from 18 up to 25 mm and length more than 20 mm (Torsello et al. 2010; Gray et al. 2017). This anatomical feature can be suitable also for iliac branch device (IBD) that in the last years has become the most popular treatment (Donas et al. 2017; Simonte et al. 2017).

The aim of our study is to retrospectively evaluate short and mid-term efficacy of standard EVAR and BBT using Endurant Medtronic (Medtronic Cardiovascular, Santa Rosa, CA, USA) stent graft in patients treated in our centre from 2009 to 2018.

## Material and methods

### Patients selection

In our study we retrospectively included all patients treated with standard EVAR combined with BBT technique using Endurant stent graft from January 1st, 2009 to December 31th, 2018.

Informed consent was obtained for all procedures. Institutional board approval was not required for this retrospective analysis.

Our series of patients had AAA with concomitant ectasic or aneurysmal CIA (Reber Type I) (Reber et al. 2001) with distal landing zone diameter from 18 mm up to 25 mm and at least 20 mm in length.

To obtain homogeneous patients group we didn’t include in the present study patients with the same anatomical features treated by other types of stent grafts (ex. Gore Excluder, etc.).

Starting from 2016 we used an IBD in case of CIAA that involved the iliac bifurcation (Reber III) with non-adequate CIA distal landing zone (shorter than 20 mm or wider than 25 mm) or in presence of internal iliac artery aneurysm.

Twenty-five patients lost to follow-up were excluded from the present study. All subjects signed the appropriate informed consent forms related to the procedure.

### Pre-operative study and endovascular procedure

A thoraco-abdominal angiography-computed tomography (CT) scan was performed before the procedures. Main grafts were oversized 10 to 20% according to aortic diameters. Oversizing of 10–15% was applied concerning iliac arteries.

All procedures were carried out in operating room equipped with C-arm Euroampli Alien 3030 (Eurocolumbus, Milan, IT). Antibiotic prophylaxis with Cefazolin (1 gr ev) was initiated prior the procedure. All patients underwent either loco-regional or general anaesthesia. An aorto-iliac angiogram was performed before device deployment. All procedures were performed by positioning Endurant stent graft according to instructions for use. A completion aorto-iliac angiogram was performed at the end of procedure to document the correct position of the endograft and patency of the hypogastric arteries.

In case of technical issues during the procedure (particularly type I endoleak), intra-operative adjunctive manoeuvres were performed.

### End points

Primary outcomes were technical success, procedural time, perioperative complications and mortality, type 1b endoleaks, 5-year aneurysm-related mortality (ARM), 5-year freedom from re-intervention.

Technical success was defined as appropriate endograft deployment, absence of type I or III endoleaks at the end of procedure, patency of renal arteries, hypogastric arteries, and femoral bifurcations.

Secondary outcomes were considered adjunctive surgical procedures, 5-year all-cause mortality and any type of endoleak occurrence during follow-up.

### Follow up

A thoraco-abdominal angiography-CT scan was obtained 1 month after the procedure. Afterwards further follow-up by ultrasound (duplex ultrasound with or without contrast medium) annually or every 6 months in case of abnormal findings. During the study period, angio-CT scan was used when surgical or endovascular re-intervention was indicated.

### Statistical analysis

Categorical variables are expressed as counts and percentages, whereas continuous variables are presented as means-standard deviation. Patient characteristics and procedural data were correlated with clinical and technical results by means of univariate analysis. The Kaplan-Meier method was used to ascertain the all-cause mortality and freedom from re-intervention.

## Results

The study was based on retrospective data routinely recorded in clinical practice. Written informed consent was obtained from patients to treat their data.

Between January 1st, 2009 and December 31th, 2018, in our Vascular Surgery Department 1690 patients underwent AAA surgical treatment. We performed 988 (58.4%) EVAR and 702 (41.6%) open surgery (OS) with progressive increasing of endovascular treatments over time.

In our study we enrolled 71 patients: 70 were treated by positioning an aorto-bi-iliac Endurant endograft, one with aorto-uniliac Endurant stent graft. The BBT was used for a total amount of 107 limbs.

They were 67 male and 4 females with mean age of 77.1 years (SD 7.7). Smoking habit and hypertension were widely present (76% and 71.8% respectively). Many patients were treated by antiplatelet therapy (83%), less by statins (54.9%). All preoperative characteristics and treatments are listed in Table 1.

**Table 1:** Preoperative characteristics and treatments

Preoperative characteristics and treatments	
Age, years	77.1 (7.7)
Male sex	67 (94.0)
DM	12 (16.9)
Smoking habit	54 (76.0)
Hypertension	51 (71.8)
COPD	12 (16.9)
Hypercholesterolemia	42.(59.1)
Antiplatelet therapy	59.(83.0)
Statin therapy	39 (54.9)
Oral anticoagulation	13 (18.3)
Beta blocker therapy	33 (46.4)

Data are mean (SD) for continuous and n (%) for categorical variables

The median aortic diameter was 56.1 mm (31.0–85.0).

The median right iliac landing zone diameter was 19.9 mm and the left one 20.3 mm. We performed 50 bell-bottom legs in the right side and 57 in the left leg. (Table 2).

**Table 2:** Anatomical features of aneurysms

Anatomical Features of Aneurysms	N	Range
AAA diameter, mm	56.1	31.0–85.0
Right CIA neck diameter, mm	19.9	10.6–57.2
Left CIA neck diameter, mm	20.3	9.1–27.2

Data are median, range

Technical success of the procedure was obtained in 100%. Two patients (2.8%) with a suspected intraoperative type 1a endoleak, were treated with an aortic cuff to achieve better infrarenal fixation in a highly tortuous aortic neck.

Some adjunctive procedures were required: 7 coils embolizations (MReye Embolization coil, Cook Medical, Bloomington, IN, USA) and overstenting in internal iliac artery aneurysm contralateral of BBT, one renal stenting in patient with severe renal artery stenosis. One patient needed an iliac plug and a femoro-femoral crossover bypass after the deployment of the aorto-uniliac stent graft. All adjunctive intraoperative procedures are listed in Table 3.

**Table 3:** Adjunctive intraoperative procedures

Adjunctive Intraoperative Procedures		%
Aortic cuff	2	2.8
Renal stenting	1	1.4
Internal iliac artery embolization	7	9.8
Bypass crossover femoro-femorol	1	1.4

The median duration of surgical procedure was 100.1 min (140.1–60.0).

There was no perioperative mortality, renal impairment or limb ischaemia.

### Follow up

The median follow-up period was 36.56 months (1–136).

At 5 years, the number of all-cause deaths was seven. 5-year ARM was not found.

**Fig. 1:**
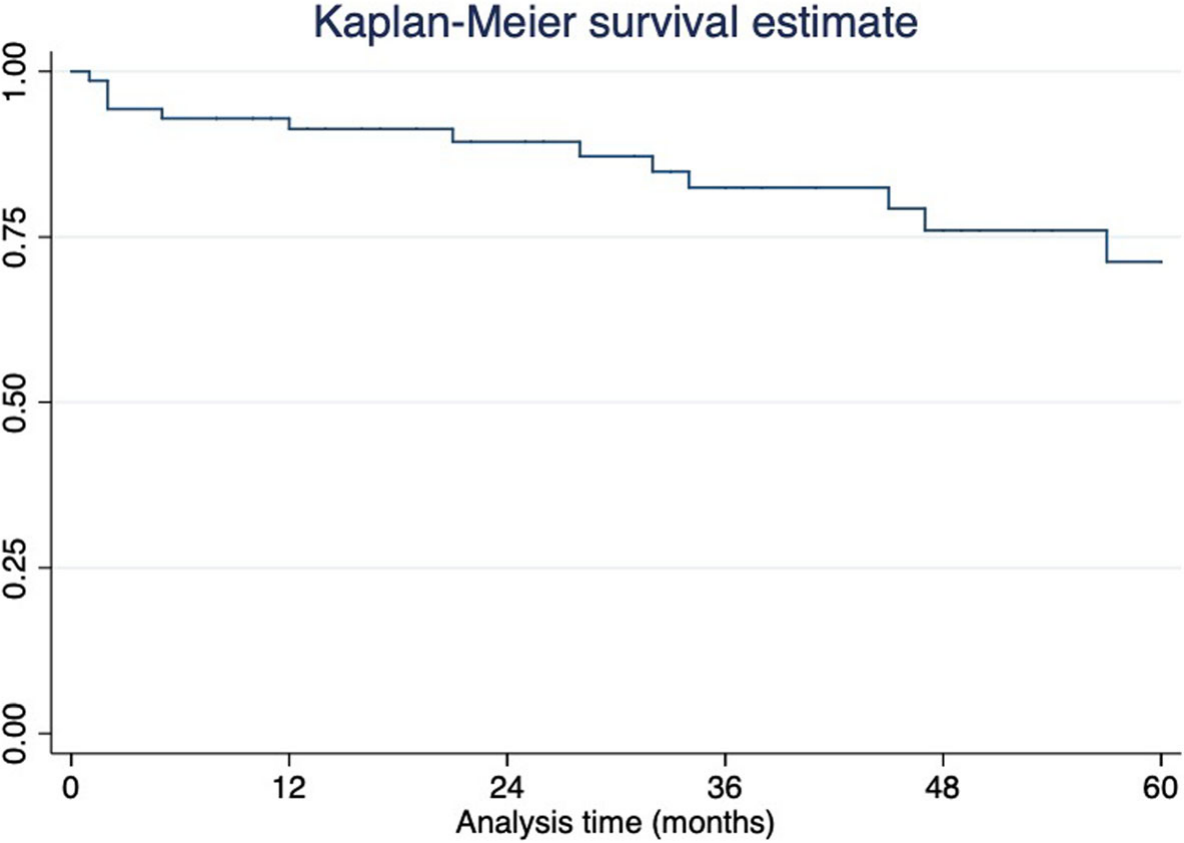
All-cause mortality according to Kaplan-Meier analysis

The 5-year overall mortality data are showed in the Kaplan Meyer table (Fig. 1).

**Fig. 2:**
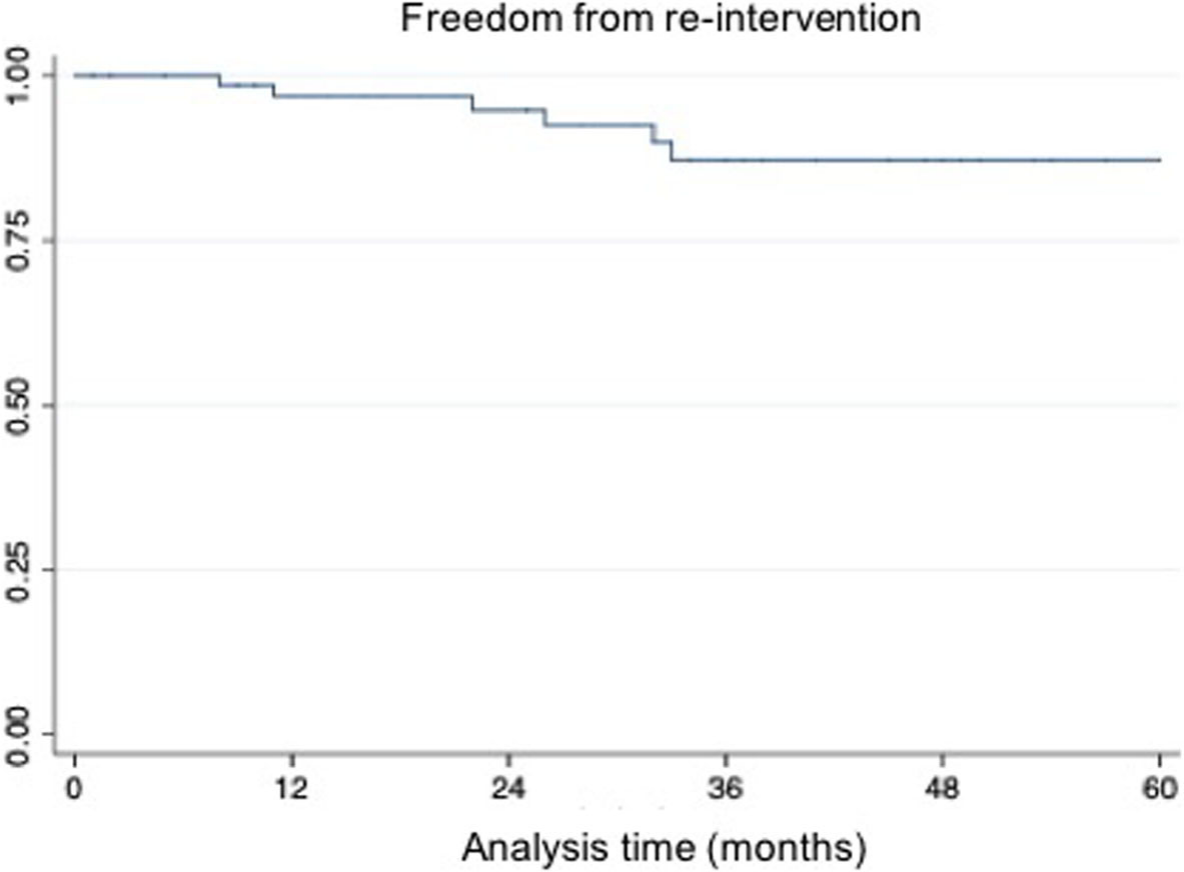
Freedom from re-intervention according to Kaplan-Meier analysis

Five-year freedom from re-intervention was 91.6%; the causes of re-interventions were types of endoleak (type 1a, 1b, 2), iliac occlusion and one IIA aneurysm. All patients were treated with endovascular technique except one with femoro-femoral crossover bypass. Freedom from re-intervention data are showed in the Kaplan Meyer table (Fig. 2).

Five-year outcomes, complications and consequent secondary procedures are listed in Tables 4 and 5, including one patient with type 1a endoleak that was operated on by aortic cuff. Type 2 endoleaks with significant increase (> 5 mm in 6 months) in aneurysmal sac diameter were detected in 2.8% of cases (2 patients); these patients were treated with repeat angiogram and selective coil embolization of lumbar arteries.

**Table 4:** 5-year follow-up outcomes

5-Year Follow-up Outcomes	N	%
All-cause mortality	13	18.3
Aneurysm related mortality	0	–
Re-interventions	6	8.4
Type 1a Endoleak	1	1.4
Type 1b Endoleak	1	1.4
Type 2 Endoleak	2	2.8
Internal iliac artery aneurysm	1	1.4
Iliac obstruction	1	1.4

**Table 5:** 5-years re-interventions

5-Years Re-interventions	N	%
**Cause of re-intervention**		
-Type 1a Endoleak	1	1.4
-Type 1b Endoleak	1	1.4
-Type 2 Endoleak	2	2.8
-Internal iliac artery aneurysm	1	1.4
-Iliac obstruction	1	1.4
**Type of re-intervention**		
Iliac extension	2	2.8
Aortic cuff	1	1.4
Lumbar arteries embolization	2	2.8
Bypass crossover	1	1.4

Five-year iliac related complications (IRC) were observed in three patients (4.4%): internal iliac artery aneurysm, iliac obstruction, type 1b endoleak. All patients were successfully treated.

Iliac arteries morphological characteristics were examined (i.e. tortuosity, calcification, length of the artery) in order to determine predictor of IRC, but we didn’t find any significant relationship.

In long term follow-up 3 others patients with type 1b endoleak at 6,7,11 years respectively from the intervention were found, all treated even though asymptomatic (endoleak was at high flow or causing aneurysmal sac growth). They were all operated on by external iliac extension and hypogastric coil embolization except one patient that underwent t an aorto-uniliac stent graft, hypogastric embolization and crossover femoro-femoral bypass. One patient died for multi organ failure after re-intervention.

## Discussion

CIA enlargement is a common finding in patient with AAA (Armon et al. 1998): up to 30% of AAAs have concomitant iliac artery aneurysm (CIAA) disease (Henretta et al. 1999). Dilatation of the iliac arteries is a risk factor for the development of type 1b endoleaks from the distal stent-graft sealing zone during endovascular treatment (Henretta et al. 1999).

Regardless of technique used, patients with CIAA have a higher re-intervention rate after EVAR compared with non-CIAA patients (Bannazadeh et al. 2017).

Several techniques have been developed to achieve the goal of sealing in CIAA, sacrificing or maintaining the hypogastric perfusion.

Following the first hypothesis the stent-graft can be extended into the external iliac artery (EIA), embolizing or simply overstenting the IIA. But IIA exclusion may cause major complications like buttock claudication, impotence, and bowel necrosis in up to 55% of the patients (Maleux et al. 2010) especially in bilateral setting.

To preserve the hypogastric perfusion, we have several opportunities: BBT, IBD, surgical replacement or endovascular revascularization of the internal iliac artery (banana technique or parallel graft, rarely described in literature often with unreported late results) (Lepidi et al. 2014).

In daily practice the most useful procedures are BBT and IBD. These techniques have different results and outcomes considering short, mid and long-term period.

BBT shows good early outcomes in term of technical success rate described higher than 97% (100% in our patients), low costs and shorter hospital stay because of low complexity of the procedure with lower amount of contrast medium and shorter operative time than IBD (Torsello et al. 2010; Pini et al. 2019; Naughton et al. 2012). In his review of 149 patients, Simonte et al. (2017) et al. revealed a mean higher procedural time compared to our (158 min vs 100) and others in literature (Donas et al. 2017).

The results are in favour of BBT also also considering the postoperative early re-interventions (Torsello et al. 2010), confirmed by our experience; instead IBD could need re-interventions for device limb occlusion or endoleak (1.6% in pELVIS registry) (Donas et al. 2017).

Also regarding mid-term outcomes, BBT shows excellent results especially in term of 1b endoleak that is the real critical issue of this procedure. Torsello et al. (2010) published in 2010 with regard of 89 patients and a mean follow-up of 56.5 months, a very low distal 1b endoleak rate (2 patients, 2.2%). Similar results are reported by Naughton in 2012: BBT was used to treat 166 CIAA limbs with a mean follow-up of 22 months with a re-intervention rate for type 1b endoleak of 4%.

The experience of this study mirrors the abovementioned findings confirming the literature data with only one patient with type 1b endoleak (1.4%) treated endovascularly.

During FU carried out by ultrasound, 5-year IRC low rate (4.4%) needing re-intervention (including type 1b endoleak, iliac occlusion or iliac dilatation) was found.

In the long period the BBT results demonstrate worst trend: Gray et al. (2017) presented 61 patients with a CIA diameter of > 20 mm with high type 1b rate endoleak (18%) compared with standard limbs (4%) with mean follow up of 53 months. Other reports (EUROSTAR database) (Griffin et al. 2015; Hobo et al. 2007) showed a high rate (9–14%) of late iliac complications in patients with BBT including type 1b endoleak and other iliac complications.

This study also found three patients with type 1b endoleak at 6, 7, 11 years respectively from the intervention confirming the critical issue about the long-term durability of this method.

However IBD has shown good late results with low re-intervention rate: pELVIS registry (Donas et al. 2017) reported 7% rate and Simonte et al. (2017) a > 90% freedom from re-intervention at 9 years.

These results suggest that BBT durability isn’t so well-defined in the long-term period with an increased type 1b endoleak rate. It advises for BBT using especially in old patients with no long-term expectancy of life.

Considering the stent graft type all studies reporting BBT are made by using various types of endografts; in our knowledge this is the first report using just one stent graft.

In our experience Medtronic Endurant stent graft demonstrated facility of use, good manageability and accurate deployment; we didn’t encounter technical problems stent graft related.

All patient with type 1b endoleak were treated by endovascular procedures confirming that BBT complications can be solved. In this series the only death type 1b endoleaks related occurred 1 month after the intervention for multi organ failure in patient with bad clinical conditions such as obesity, severe COPD and chronic ischaemic heart disease.

These results underline that patients treated by BBT need a careful FU to detect and quickly treat any type of procedural related problems.

Despite our study has some limitations due to be a single-centre and single-brand report, our data show that Endurant stent graft represents a good solution for the treatment of wide or aneurysmal CIA with adequate distal landing zone.

## Conclusions

According with the results of this study it seems that BBT is easy to perform and does not increase the invasiveness of the procedure. It is an effective treatment with good short and mid-term results, characterized by a low rate of IRC and no ARM. Immediate and mid-term complications and secondary procedures rates are as rare as IBD ones. More data are required to clarify the BBT durability in long-term period.

## Data Availability

The datasets used and analysed during the current study are available from the corresponding author on reasonable request.

## Abbreviations

EVAR: Endovascular aneurysm repair
CIA: Common iliac artery
IIA: Internal iliac artery
BBT: Bell-bottom technique
AAA: Abdominal aortic aneurysm
CT: Computed tomography
ARM: Aneurysm related mortality
IRC: Iliac related complication
CIAA: Common iliac artery aneurysm
IBD: Iliac branch device

## References

[CR1] Armon MP, Wenham PW, Whitaker SC (1998). Common iliac artery aneurysms in patients with abdominal aortic aneurysms. Eur J Vasc Endovasc Surg.

[CR2] Bannazadeh M, Jenkins C, Forsyth A (2017). Outcomes for concomitant common iliac artery aneurysms after endovascular abdominal aortic aneurysm repair. J Vasc Surg.

[CR3] Donas KP, Inchingolo M, Cao P (2017). Secondary procedures following iliac branch device treatment of aneurysms involving the iliac bifurcation: the pELVIS registry. J Endovasc Ther.

[CR4] Gray D, Shahverdyan R, Reifferscheid V (2017). EVAR with flared iliac limbs has a high risk of late type 1b Endoleak. Eur J Vasc Endovasc Surg.

[CR5] Griffin CL, Scali ST, Feezor RJ (2015). Fate of aneurysmal common iliac artery landing zones used for endovascular aneurysm repair. J Endovasc Ther.

[CR6] Henretta JP, Karch LA, Hodgson KJ (1999). Special iliac artery considerations during aneurysm endografting. Am J Surg.

[CR7] Hobo R, Laheij RJ, Buth J (2007). The influence of aortic cuffs and iliac limb extensions on the outcome of endovascular abdominal aortic aneurysm repair. J Vasc Surg.

[CR8] Lepidi S, Piazza M, Scrivere P (2014). Parallel endografts in the treatment of distal aortic and common iliac aneurysms. Eur J Vasc Endovasc Surg.

[CR9] Maleux G, Willems E, Vaninbroukx J (2010). Outcome of proximal internal iliac artery coil embolization prior to stent-graft extension in patients previously treated by endovascular aortic repair. J Vasc Interv Radiol.

[CR10] Massière B, Von Ristow A, Vescovi A (2016). Endovascular therapeutic options for the treatment of aorto-iliac aneurysms. Rev Col Bras Cir.

[CR11] Naughton P, Park MS, Kheirelseid AH (2012). A comparative study of the bell-bottom technique vs hypogastric exclusion for the treatment of aneurysmal extension to the iliac bifurcation. J Vasc Surg.

[CR12] Pini R, Faggioli G, Indelicato G (2019). Early and late outcome of common iliac aneurysms treated by flared limbs or iliac branch devices during endovascular aortic repair. JVIR.

[CR13] Reber PU, Brunner K, Hakki H (2001). Klassifikation und Therapie der isolierten Beck- enarterienaneurysmen [incidence, classification and therapy of isolated pelvic artery aneurysm]. Chirurg..

[CR14] Simonte G, Parlani G, Farchioni L (2017). Lesson Learned with the Use of Iliac Branch Devices: Single Centre 10 Year Experience in 157 Consecutive Procedures. Eur J Vasc Endovasc Surg.

[CR15] Torsello G, Schonefeld E, Osada N (2010). Endovascular treatment of common iliac artery aneurysms using the bell-bottom technique: long-term results. J Endovasc Ther.

[CR16] Wanhainen A, Verzini F, Van Herzeele I (2019). European Society for Vascular Surgery (ESVS) 2019. Clinical practice guidelines on the management of abdominal aorto-iliac artery aneurysms. Eur J Vasc Endovasc Surg.

